# Intravoxel Incoherent Motion Diffusion-Weighted Imaging for Quantitative Differentiation of Breast Tumors: A Meta-Analysis

**DOI:** 10.3389/fonc.2020.585486

**Published:** 2020-10-20

**Authors:** Jianye Liang, Sihui Zeng, Zhipeng Li, Yanan Kong, Tiebao Meng, Chunyan Zhou, Jieting Chen, YaoPan Wu, Ni He

**Affiliations:** Department of Medical Imaging, Sun Yat-sen University Cancer Center, State Key Laboratory of Oncology in South China, Collaborative Innovation Center for Cancer Medicine, Guangzhou, China

**Keywords:** breast tumors, post-test probability, intravoxel incoherent motion diffusion-weighted imaging, differential diagnosis, meta-analysis

## Abstract

**Objectives:** The diagnostic performance of intravoxel incoherent motion diffusion–weighted imaging (IVIM-DWI) in the differential diagnosis of breast tumors remains debatable among published studies. Therefore, this meta-analysis aimed to pool relevant evidence regarding the diagnostic performance of IVIM-DWI in the differential diagnosis of breast tumors.

**Methods:** Studies on the differential diagnosis of breast lesions using IVIM-DWI were systemically searched in the PubMed, Embase and Web of Science databases in recent 10 years. The standardized mean difference (SMD) and 95% confidence intervals of the apparent diffusion coefficient (ADC), tissue diffusivity (D), pseudodiffusivity (D^*^), and perfusion fraction (f) were calculated using Review Manager 5.3, and Stata 12.0 was used to pool the sensitivity, specificity, and area under the curve (AUC), as well as assess publication bias and heterogeneity. Fagan's nomogram was used to predict the posttest probabilities.

**Results:** Sixteen studies comprising 1,355 malignant and 362 benign breast lesions were included. Most of these studies showed a low to unclear risk of bias and low concerns regarding applicability. Breast cancer had significant lower ADC (SMD = −1.38, *P* < 0.001) and D values (SMD = −1.50, *P* < 0.001), and higher f value (SMD = 0.89, *P* = 0.001) than benign lesions, except D^*^ value (SMD = −0.30, *P* = 0.20). Invasive ductal carcinoma showed lower ADC (SMD = 1.34, *P* = 0.01) and D values (SMD = 1.04, *P* = 0.001) than ductal carcinoma *in situ*. D value demonstrated the best diagnostic performance (sensitivity = 86%, specificity = 86%, AUC = 0.91) and highest post-test probability (61, 48, 46, and 34% for D, ADC, f, and D^*^ values) in the differential diagnosis of breast tumors, followed by ADC (sensitivity = 76%, specificity = 79%, AUC = 0.85), f (sensitivity = 80%, specificity = 76%, AUC = 0.85) and D^*^ values (sensitivity = 84%, specificity = 59%, AUC = 0.71).

**Conclusion:** IVIM-DWI parameters are adequate and superior to the ADC in the differentiation of breast tumors. ADC and D values can further differentiate invasive ductal carcinoma from ductal carcinoma *in situ*. IVIM-DWI is also superior in identifying lymph node metastasis, histologic grade, and hormone receptors, and HER2 and Ki-67 status.

## Introduction

Breast cancer is one of the most common malignant tumors and the second leading cause of cancer death in females (1). Early detection and accurate diagnosis of breast cancer with various histological/molecular subtypes, such as estrogen receptor (ER), progesterone receptor (PR), human epidermal growth factor receptor 2 (HER2), and Ki-67 proliferation indexes, are helpful for developing individualized therapies and achieving a better prognosis. Screening the breast lesions with conventional mammography is challenging for clinician due to the low sensitivity in dense breast parenchyma (2). Dynamic contrast-enhanced magnetic resonance imaging (DCE-MRI) is a common MRI sequence in clinical practice, which can reflect the morphological and haemodynamic features of breast lesions. A previous meta-analysis which included studies using DCE-MRI as an adjunct to conventional mammography or ultrasound to clarify uncertain finding without microcalcification, demonstrated that breast MRI had an excellent diagnostic performance with a pooled sensitivity of 99% and specificity of 89% (3). However, the specificity is still variable due to background parenchymal enhancement and overlapped kinetic enhancement patterns between breast cancer and benign lesion. The false-positive findings may cause additional examination or unnecessary surgery (4).

Diffusion-weighted imaging (DWI) has become a promising technique in the differential diagnosis of breast lesions, which allows measurement of water molecular movement using apparent diffusion coefficient (ADC) values. The international European Society of Breast Imaging (EUSOBI) working group has confirmed the importance of breast DWI in the multiparametric breast MRI protocol to differentiate between breast cancer and benign lesions, distinguish *in situ* from invasive lesions, and predict the responses to neoadjuvant therapy over time (5). Breast cancer usually has high cellularity (low diffusivity) and high vascularity (high perfusion), which may impact ADC values in a diametrically opposite direction.

Intravoxel incoherent motion (IVIM) is an advanced imaging technique that was first proposed by Le Bihan et al. (6). This procedure can distinguish the incoherent motion of water molecules within the capillaries from molecular diffusion in the extravascular space (7). The true diffusion coefficient (D value), pseudodiffusion coefficient (D^*^ value) and perfusion fraction (f value) were generated using a biexponential model with multiple *b*-values (8). Several studies have applied IVIM-DWI to discriminate breast cancer from benign breast lesions and characterize the histological/molecular subtypes of breast cancer better diagnostic performance than traditional ADC values (7, 9, 10). However, the diagnostic performance of IVIM-DWI-derived parameters in the differentiation of breast tumors is not consistent, and the application of this sequence remains debatable. For example, several studies (7, 11, 12) indicated that breast cancer had a higher D^*^ value than did benign lesions, while other studies reported adverse (10, 13–15) or non-significant results (9, 16, 17). The studies of Cho et al. (9) and Lin et al. (13) suggested that the IVIM model can further distinguish invasive ductal carcinoma (IDC) from ductal carcinoma *in situ* (DCIS), while another study reported no significant difference in the D, D^*^, and f values between them (7). Last but not least, the small sample sizes in most studies were still insufficient to draw a robust conclusion for the performance of IVIM-DWI; therefore, clinical guidelines for the application in the breast have not been established. To address this problem, we perform a meta-analysis of all the published results regarding the diagnostic performance of IVIM-DWI in differentiating malignant and benign breast lesions. The controversial issues among the different studies will be addressed with more reliable evidence.

## Materials and Methods

### Data Sources

Studies on the differential diagnosis of breast tumors using IVIM-DWI parameters published in the past 10 years were systemically retrieved from PubMed, Embase and Web of Science by two senior librarians. A search formula was created using different combinations of medical subject headings or key words related to the following terms: IVIM, intravoxel incoherent motion, multiple *b*-values DWI, biexponential, true diffusion coefficient, pseudodiffusion coefficient, perfusion fraction, and breast or breast lesion/cancer/carcinoma. We also performed manual retrieval of the reference lists from the included studies.

### Study Selection

Studies that met the following criteria were included: (a) the research purpose was to differentiate malignant and benign breast lesions using IVIM-DWI parameters; (b) the mean and standard deviation (SD) of each parameter were provided; (c) the diagnostic performance regarding sensitivity and specificity, or true-positive (TP), false-negative (FN), false-positive (FP), and true-negative (TN) counts were reported; and (d) breast cancer was confirmed by pathology after initial MRI examination. Exclusion criteria mainly included (a) duplication from the same authors or institutions; (b) meta-analysis, conference abstract, review or any unpublished results; (c) animal experiments or studies not on breasts; (d) non-English studies; and (e) studies with *b*-values >2,500 s/mm^2^, to maximally avoid non-Gaussian diffusion.

### Data Extraction

A spreadsheet was used to extract the mean values and SDs as well as the diagnostic performance of the ADC, D, D^*^, and f values with threshold value, area under the curve (AUC), sensitivity, specificity or the TP, FN, FP, and TN in the respective study by one author and reviewed by another author. Other information included first author, publication year, country, field strength and vendors, *b*-values, patient ages, tumor size, and published journals.

### Quality Assessment

The quality of the studies and likelihood of bias were evaluated using Review Manager 5.3 software (Cochrane Collaboration), referring to the Quality Assessment of Diagnostic Accuracy Studies-2 (18). We assessed the risk of bias and applicability in four domains: patient selection, index tests, reference standards, and flow and timing (19).

### Publication Bias and Heterogeneity Evaluation

Because two parts of the data were pooled in our study—quantitative values and the diagnostic performance of each parameter, funnel plots and Begg's test were used to visually and quantitatively assess the publication bias for the continuous variables, whereas Deek's plot was used to assess the publication bias of the sensitivity and specificity with Stata version 12.0 (StataCorp). For an asymmetric or skewed funnel plot, *P* < 0.05 in Begg's test or Deeks' test, indicated the potential of publication bias (20). The inconsistency index (*I*^2^) and Cochran's *Q*-tests were used to explore the heterogeneity of the included studies, with *I*^2^ >50% or *P* < 0.05 for the Cochran *Q*-test suggesting statistically significant heterogeneity; in these instances, a random-effect model was applied for subsequent pooling, or a fixed-effect model when *I*^2^ < 50% (21).

### Data Synthesis

We constructed forest plots for continuous variables and calculated the standardized mean difference (SMD) between malignant and benign breast lesions using Review Manager 5.3 software. We used the bivariate mixed-effects binary regression model in Stata version 12.0 to pool the diagnostic performance with sensitivity, specificity, positive likelihood ratio (PLR), negative likelihood ratio (NLR), diagnostic odds ratio (DOR), and AUC. The summary receiver operating characteristic (SROC) curves and Fagan's nomograms were also plotted to determine the diagnostic values and predict the post-test probabilities of the ADC, D, D^*^ and f values in obtaining a differential diagnosis of breast tumors. Meta-disc 1.4 was used to evaluate the threshold effects by calculating the Spearman correlation coefficient (r) between the logit (TP rate) and logit (FP rate).

## Results

### Literature Search and Selection

**Figure 1:**
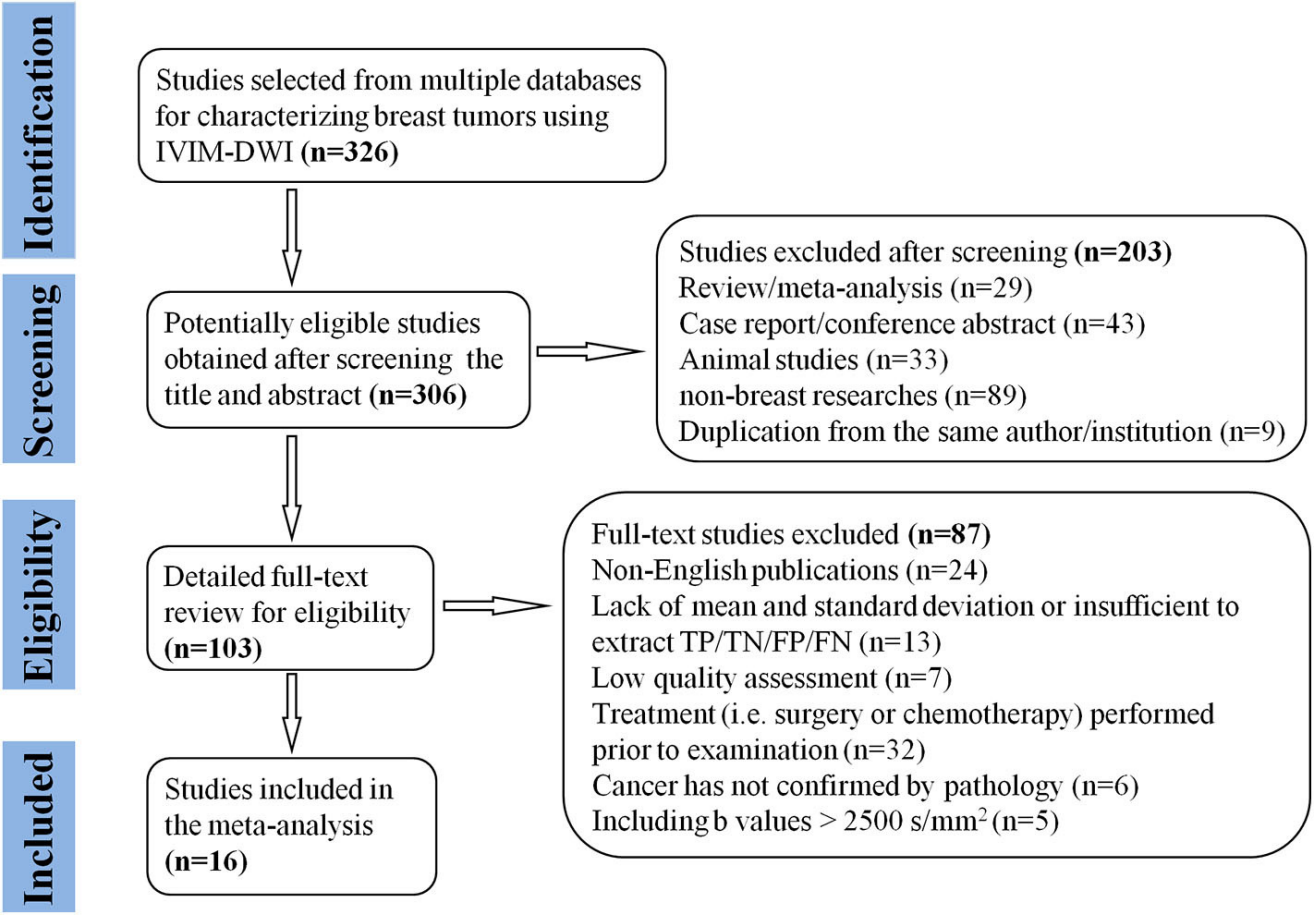
Flowchart detailing the study selection process. Sixteen studies met the inclusion criteria. FN, false negative; FP, false positive; TN, true negative; TP, true positive.

A flowchart detailing the study selection process is provided in Figure 1. Although the study by Iima et al. (22) included *b*-values of 2,000 and 2,500 s/mm^2^ which may induce non-Gaussian diffusion, they used a hybrid model to sufficiently separate the non-Gaussian diffusion from IVIM effects. We also performed a sensitivity analysis and compared the pooled results between before and after excluding the study, the results were not significantly changed. Therefore, we considered the study by Iima et al. is suitable to be included. Sixteen eligible studies with 1,355 malignant and 362 benign breast lesions were included for analysis. Basic information and diagnostic performance for each included study are detailed in Tables 1, 2. The breast cancer subtypes mainly included DCIS, IDC, lobular carcinoma *in situ*, invasive lobular carcinoma, intraductal papillary carcinoma, squamous cell carcinoma, mucinous carcinoma and malignant phyllodes tumors. Benign lesions consisted of fibroadenoma, intraductal papilloma, granulomatous mastitis, epithelial proliferative lesion, fibrocystic change, and phyllodes tumors.

**Table 1:** Basic information from each included study.

**Author**	**Year**	**Country**	**Machine type**	***b*-values (s/mm^**2**^)**	**Age (years)**	**Tumor diameters (mm)**	**Malignant**	**Benign**	**Journal**
Bokacheva et al. (23)	2013	USA	3T GE	0, 30, 60, 90, 120, 400, 600, 800, 1,000	49 (28–70)	Benign: 20 (8–48); Malignant: 38 (9–80)	26	14	J Magn Reson Imaging
Chen et al. (24)	2017	China	3T Siemens	0, 50, 100, 150, 200, 300, 400, 800, 1,000	47 (15–62)	Malignant: 102 mm^2^; Benign: 78.37 mm^2^	18	11	J Appl Clin Med Phys
Cho et al. (9)	2016	USA	3T Siemens	0, 30, 70, 100, 150, 200, 300, 400, 500, 800	Benign: 46.3 ± 11.7; Malignant: 50.2 ± 10.5	32.5 ± 27.2	50	12	Eur Radiol
Jiang et al. (25)	2017	China	3T GE	0, 10, 30, 50, 70, 100, 150, 200, 400, 600, 1,000, 1,500	45 ± 10	Malignant: 30.5 ± 3.8; Benign: 22.9 ± 4.2	31	35	J Comput Assist Tomogr
Iima et al. (22)	2017	Japan	3T Siemens	5, 10, 20, 30, 50, 70, 100, 200, 400, 600, 800, 1,000, 1,500, 2,000, 2,500	58.5 (20–88)	Benign: 25.7 (10–100); Malignant: 18.2 (10–62)	152	47	Radiology
Lin et al. (13)	2017	China	3T Philips	0, 50, 100, 150, 200, 500, 800	48 (17–77)	–	51	47	Int J Clin Exp Med
Liu et al. (14)	2016	China	1.5T Philips	0, 10, 20, 30, 50, 70, 100, 150, 200, 400, 600, 1,000	NA	Malignant: 28.32 ± 4.25; Benign: 22.27 ± 3.96	36	23	Eur Radiol
Ma et al. (11)	2017	China	3T Siemens	0, 50, 100, 150, 200, 250, 300, 400, 600, 800, 1,000, 1,200	48.2 ± 5.1	NA	81	47	Magn Reson Imaging
Wang et al. (15)	2016	China	3T GE	0, 10, 20, 50, 100, 200, 300, 400, 600, 800	46.85 ± 8.63	Malignant: 159.9 (82.6–243.2) mm^2^; Benign: 87.5 (55.3–189.7) mm^2^	31	23	Breast Care
Zhao et al. (10)	2018	China	3T GE	0, 50, 100, 150, 200, 400, 500, 1,000, 1,500	Benign: 46.3 ± 11.7; Malignant: 50.2 ± 10.5	NA	119	22	Oncol Lett
Kim et al. (17)	2016	Korea	3T Philips	0, 30, 70, 100, 150, 200, 300, 400, 500, 800	51 (28–83)	20 (10–62)	275	275	Br J Radiol
Lee et al. (26)	2016	Korea	3T Siemens	0, 25, 50, 75, 100, 150, 200, 300, 500, 800	53 (34–77)	10–66	82	0	J Magn Reson Imaging
Dijkstra et al. (27)	2015	Netherlands	1.5T Siemens	0, 50, 200, 500, 800, 1,000	47 (22–75)	NA	116	23	J Magn Reson Imaging
Kawashima et al. (28)	2017	Japan	3T GE	0, 20, 40, 80, 120, 200, 400, 600, 800	58 (32–85)	20 (10–75)	137	0	Acad Radiol
Meng et al. (29)	2020	China	3T GE	0, 50, 75, 100, 150, 200, 400, 800, 1,000	Benign: 41 ± 12; Malignant: 58 ± 10	Malignant: 25.6 ± 11.4; Benign: 22.4 ± 8.9	65	58	J Magn Reson Imaging
Song et al. (30)	2019	Korea	3T Siemens	0, 10, 20, 30, 50, 70, 100, 150, 200, 400, 600, 1,000	54 (35–81)	18 (8–48)	85	0	J Magn Reson Imaging

*NA, not available*.

**Table 2:** Diagnostic performance of each included study.

	**Author**	**Year**	**Threshold**	**AUC**	**Sensitivity**	**Specificity**	**TP**	**FP**	**FN**	**TN**
ADC	Bokacheva et al. (23)	2013	1.54	0.72	0.65	0.71	17	4	9	10
	Cho et al. (9)	2016	NA	0.69	0.58	0.833	29	2	21	10
	Lin et al. (13)	2017	1.203	0.931	0.894	0.843	46	7	5	40
	Wang et al. (15)	2016	NA	NA	0.808	0.677	46	14	11	30
	Zhao et al. (10)	2018	1.15	0.9	0.857	0.893	63	2	17	20
D	Bokacheva et al. (23)	2013	1.52	0.75	0.85	0.64	22	5	4	9
	Cho et al. (9)	2016	NA	0.77	0.66	0.917	33	1	17	11
	Lin et al. (13)	2017	1.096	0.945	0.872	0.843	44	7	7	40
	Liu et al. (14)	2016	1.02	0.917	0.89	0.83	32	4	4	19
	Wang et al. (15)	2016	NA	NA	0.937	0.874	53	6	4	38
	Meng et al. (29)	2020	1.01	0.809	0.7385	0.9138	48	5	17	53
	Zhao et al. (10)	2018	1.09	0.92	0.929	0.88	111	3	8	19
D*	Bokacheva et al. (23)	2013	0.58	0.84	0.85	0.86	22	2	4	12
	Cho et al. (9)	2016	NA	0.5	1	0.25	50	9	0	3
	Lin et al. (13)	2017	99.056	0.682	0.702	0.588	36	19	15	28
	Liu et al. (14)	2016	140.88	NA	0.86	0.74	31	6	5	17
	Meng et al. (29)	2020	26.58	0.67	0.7385	0.6207	48	22	17	36
	Zhao et al. (10)	2018	43.18	0.674	0.714	0.547	85	10	34	12
f	Bokacheva et al. (23)	2013	4.9	0.79	0.73	0.86	19	2	7	12
	Cho et al. (9)	2016	NA	0.72	0.833	0.726	42	3	8	9
	Lin et al. (13)	2017	7.87	0.802	0.863	0.66	44	16	7	31
	Liu et al. (14)	2016	7.2	NA	0.86	0.74	31	6	5	17
	Meng et al. (29)	2020	4.99	0.766	0.7385	0.7586	48	14	17	44
	Zhao et al. (10)	2018	20.3	0.885	0.857	0.893	50	2	17	20

NA, not available; ADC, apparent diffusion coefficient; D, tissue diffusivity, D*, pseudo-diffusivity; f, perfusion fraction; AUC, area under the curve; FN, false negative, FP, false positive; TN, true negative, TP, true positive. Threshold values of ADC, D and D* are factors of 10^−3^ mm^2^/s.

### Quality Assessment

**Figure 2:**
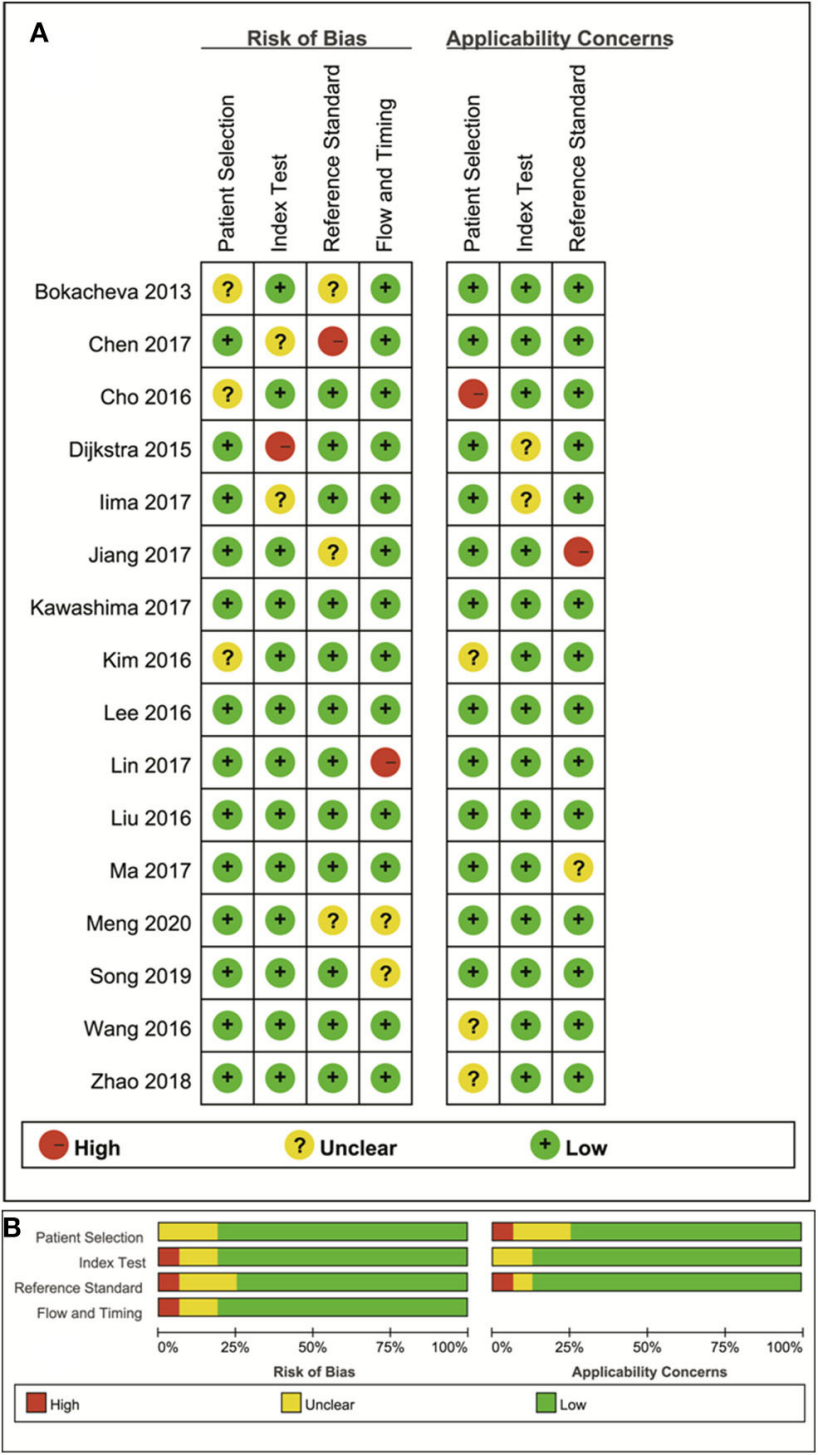
The distribution of risk of bias and applicability concerns for each included study using Quality Assessment of Diagnostic Accuracy Studies-2 (QUADAS-2) **(A)** and a summary of the methodological quality **(B)**.

The distribution of the Quality Assessment of Diagnostic Accuracy Studies−2 scores for risk of bias and applicability concerns are shown in Figure 2. The overall quality of the included studies was acceptable. In the patient selection domain, six studies showed unclear risk of bias due to ambiguity regarding patient enrolment and study design. The applicability concerns remained unclear or high in five studies, because the malignant and benign tumor types were inconsistent between studies. Four studies were marked unclear risk of bias with high concerns of applicability for the index test domain, because the threshold values for ADC, D, D^*^ or f were not provided. Six studies showed unclear or high risks of bias in reference standards domain because some of the benign lesions were diagnosed via long-term follow-up. Most studies had a low risk of bias regarding patient flow and timing domains because of the short time interval between MR examination and pathological confirmation (within 1 week).

### Quantitative Analysis

#### ADC Used for Diagnosis of Breast Tumor

**Figure 3:**
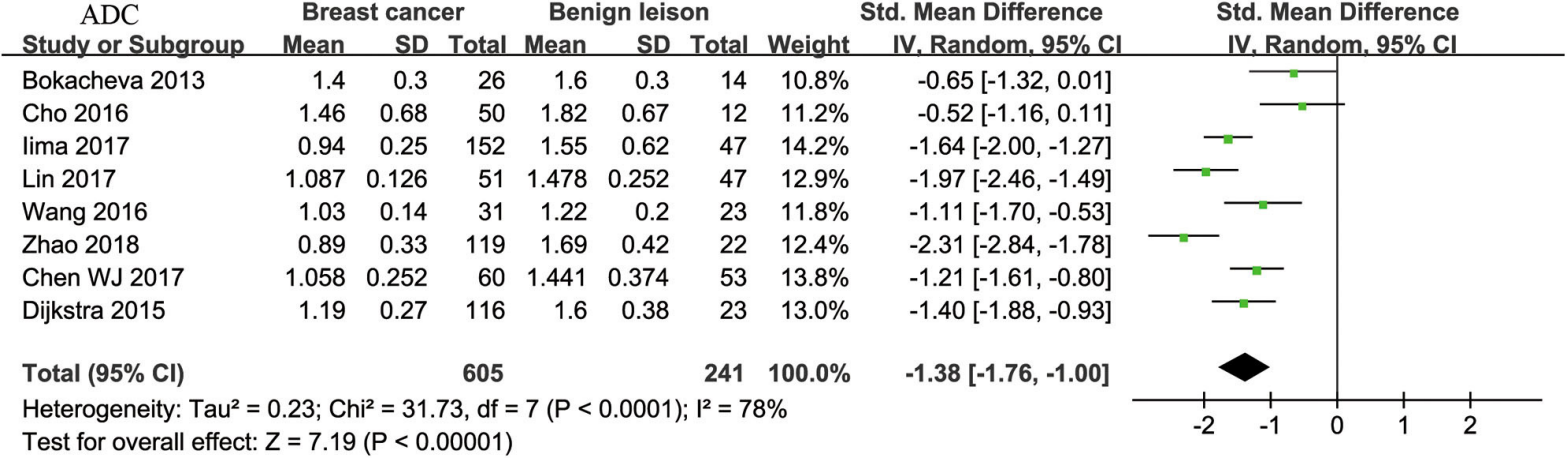
Forest plot of the mean value of the apparent diffusion coefficient (ADC) between malignant and benign breast lesions. The standardized mean differences (SMDs) indicated that breast cancers had a significantly lower ADC than benign lesions.

Eight studies regarding ADC used in differentiating breast tumors were included for analysis. The χ^2^ = 31.73 and *P* < 0.001 of the heterogeneity test (*I*^2^ = 78%) suggested high heterogeneity among the included studies. The forest plot in Figure 3 shows the distribution of the ADC between malignant and benign breast lesions. A random-effects model generated an SMD of −1.38 (−1.76, −1.00) (*P* < 0.001) between malignant and benign breast lesions for ADC. The Begg's test suggested no publication bias relating to the ADC (*P* = 0.428).

#### D Value Used for Diagnosis of Breast Tumor

**Figure 4:**
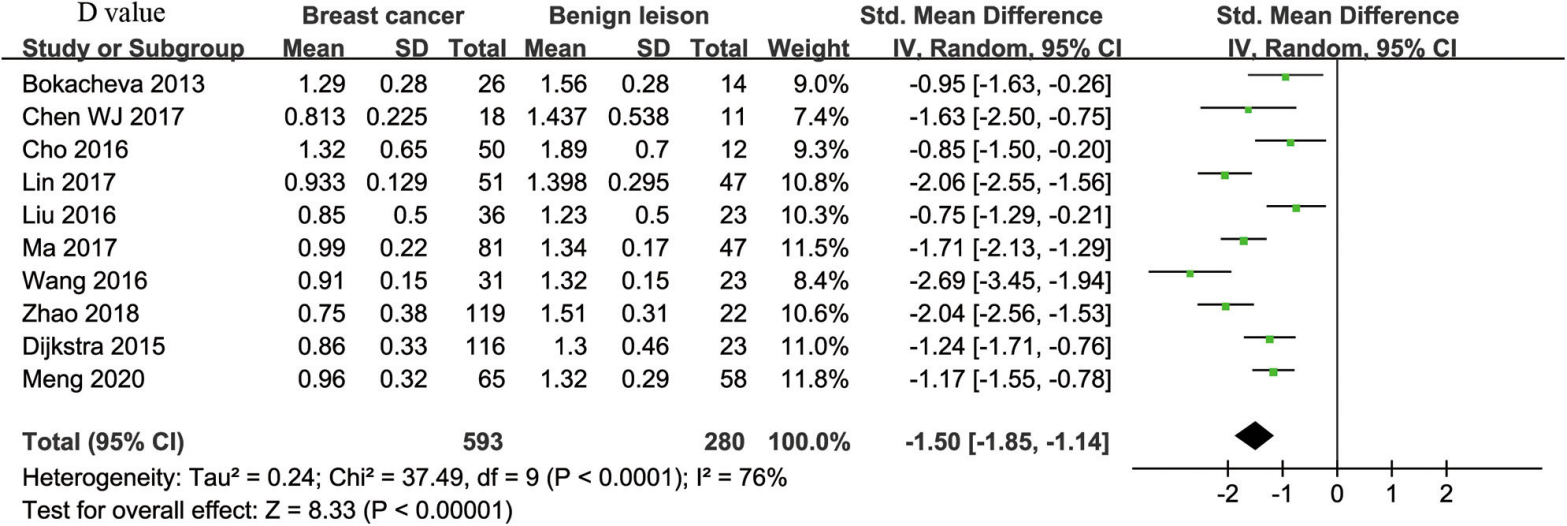
Forest plot of the mean value of tissue diffusivity (D) between malignant and benign breast lesions. The standardized mean differences (SMDs) indicated that breast cancers had a significantly lower D value than did benign lesions.

Ten studies regarding D value used in differentiating breast tumors were included for analysis. The χ^2^ = 37.49 and *P* < 0.001 of the heterogeneity test (*I*^2^ = 76%) suggested high heterogeneity among the included studies. The forest plot in Figure 4 shows the distribution of D between malignant and benign breast lesions. A random-effects model generated an SMD of −1.50 (−1.85, −1.14) (*P* < 0.001) between malignant and benign breast lesions for D. The Begg's Test suggested no publication bias relating to D (*P* = 0.112).

#### D^*^ Value Used for Diagnosis of Breast Tumor

**Figure 5:**
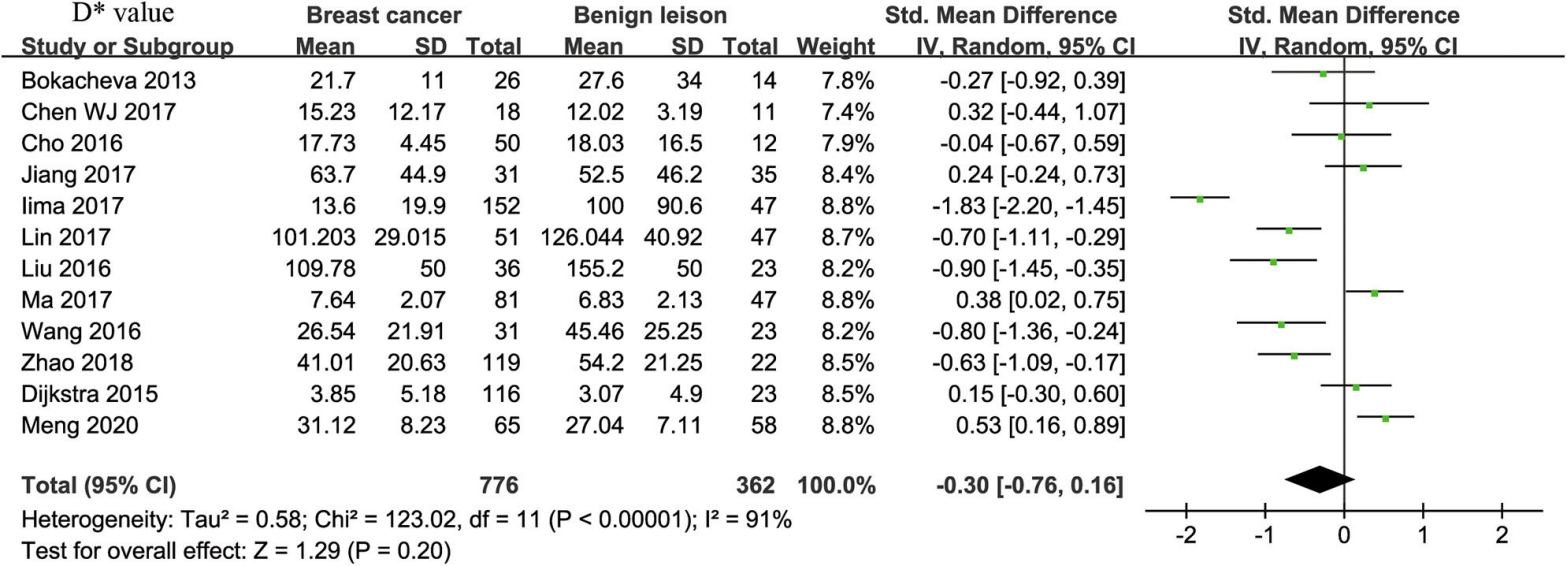
Forest plot of the mean value of pseudodiffusivity (D*) between malignant and benign breast lesions. The standardized mean differences (SMDs) indicated that there is no statistical difference between breast cancers and benign lesions in D* value.

Twelve studies regarding D^*^ value used in differentiating breast tumors were included for analysis. The χ^2^ = 123.02 and *P* < 0.001 of the heterogeneity test (*I*^2^ = 91%) suggested high heterogeneity among the included studies. The forest plot in Figure 5 shows the distribution of D^*^ between malignant and benign breast lesions. A random-effects model generated an SMD of −0.30 (−0.76, 0.16) (*P* = 0.20) between malignant and benign breast lesions for D^*^. The Begg's test suggested no publication bias relating to D^*^ (*P* = 0.208).

#### f-Value Used for Diagnosis of Breast Tumor

**Figure 6:**
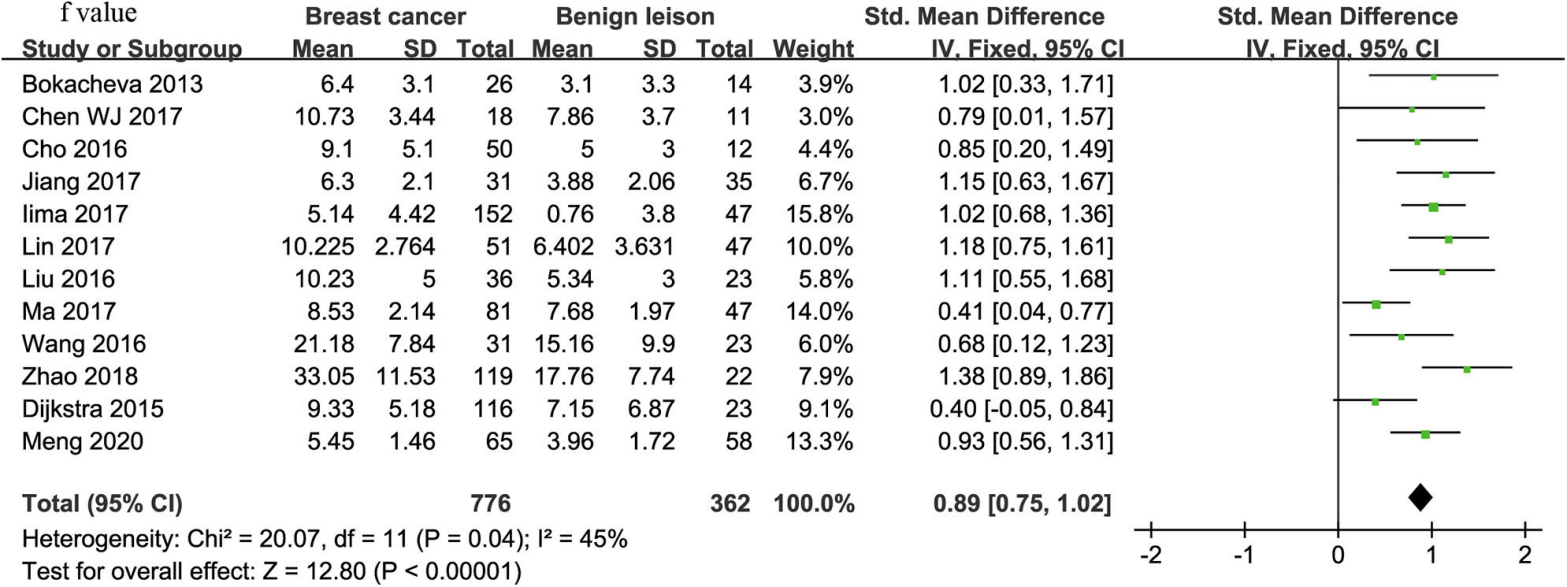
Forest plot of the mean value of perfusion fraction (f) between malignant and benign breast lesions. The standardized mean differences (SMDs) indicated that breast cancers had a significantly higher f value than did benign lesions.

Twelve studies regarding f value used in differentiating breast tumors were included for analysis. The χ^2^ = 20.07 and *P* = 0.04 of the heterogeneity test (*I*^2^ = 45%) suggested mild heterogeneity among the included studies. The forest plot in Figure 6 shows the distribution of f between malignant and benign breast lesions. A fixed-effects model generated an SMD of 0.89 (0.75, 1.02) (*P* < 0.001) between malignant and benign breast lesions for f value. The Begg's test suggested no publication bias in f (*P* = 0.880).

#### Subgroup Analysis for Histological/Molecular Subtypes

Because the treatment strategy and prognosis were different between DCIS and IDC, and because several studies have provided differential information between DCIS and IDC as well as other pathologic prognostic factors such as tumor size, lymph node metastasis, histologic grade, and the molecular expression of ER, PR, HER2, and Ki-67 in breast cancer, we further pooled these results. The pooled results were listed in Table 3. The results suggested that IDC had lower ADC (SMD = 1.34, *P* = 0.01) and D values (SMD = 1.04, *P* = 0.001) than did DCIS. No significant difference was observed in the ADC, D, D^*^, and f values between tumors ≥2 cm and those <2 cm (all *P* ≥ 0.05). Higher D^*^ (SMD = −0.23, *P* = 0.009) and f values (SMD = −0.28, *P* = 0.001) were observed in lesions with metastatic lymph nodes comparing to lesions with negative lymph nodes metastasis. HER2-positive cancer showed higher D^*^ (SMD = −0.28, *P* = 0.003) and f values (SMD = −0.24, *P* = 0.009) than did HER2-negative cancer. Tumors with high Ki-67 expression had a lower D value than those with low Ki-67 expression (SMD = 0.26, *P* = 0.002). There was also a significant difference in the D^*^ value between ER-positive and ER-negative tumors, PR-positive and PR-negative tumors, and low-grade and high-grade tumors (all *P* < 0.05).

**Table 3:** Differential information between DCIS and IDC and the pathologic prognostic factors.

**Included studies**	**Groups**	**Number**	**ADC**			**D**			D*			**f**		
			**SMD**	***P***	***I*^**2**^**	**SMD**	***P***	***I*^**2**^**	**SMD**	***P***	***I*^**2**^**	**SMD**	***P***	***I*^**2**^**
Subtypes, *n* = 2	DCIS	15	1.34 (0.28, 2.41)	**0.01**	67%	1.04 (0.46, 1.62)	**0.001**	0	0.23 (−0.33, 0.79)	0.42	0	−0.41 (−0.97, 0.15)	0.15	0
(9, 13)	IDC	70												
Estrogen, *n* = 6	Negative	177	0.18 (−0.25, 0.61)	0.40	79%	−0.15 (−0.84, 0.54)	0.67	93%	0.45 (0.01, 0.89)	**0.04**	82%	0.12 (−0.05, 0.29)	0.17	47%
(9, 10, 17, 26, 29, 30)	Positive	429												
Progesterone, *n* = 6	Negative	273	−0.02 (−0.46, 0.41)	0.92	82%	−0.04 (−0.53, 0.45)	0.88	88%	0.68 (0.51, 0.85)	**0.001**	89%	0 (−0.16, 0.16)	1	0
(9, 10, 17, 26, 29, 30)	Positive	398												
Tumor size, *n* = 4	<2 cm	266	−0.02 (−0.20, 0.17)	0.87	0	0.02 (−0.15, 0.20)	0.79	0	−0.33(−0.68, 0.03)	0.07	70%	−0.10 (−0.28, 0.07)	0.26	0
(17, 26, 29, 30)	≥ 2 cm	241												
Lymph node, *n* = 5	Negative	376	−0.06 (−0.25, 0.12)	0.49	39%	0.10 (−0.22, 0.43)	0.53	67%	−0.23 (−0.40, −0.06)	**0.009**	46%	−0.28 (−0.46, −0.11)	**0.001**	84%
(10, 17, 26, 29, 30)	Positive	250												
Histologic grade, *n* = 4	Grades 1, 2	262	−0.11 (−0.30, 0.07)	0.23	27%	−0.07 (−0.42, 0.28)	0.69	67%	−0.47 (−0.93, −0.01)	**0.04**	81%	0.03 (−0.15, 0.21)	0.76	0
(17, 26, 29, 30)	Grade 3	232												
HER2, *n* = 5	Negative	455	−0.15 (−0.34, 0.04)	0.12	32%	−0.06 (−0.23, 0.12)	0.55	46%	−0.28 (−0.46, −0.10)	**0.003**	72%	−0.24 (−0.43, −0.06)	**0.009**	92%
(10, 17, 26, 29, 30)	Positive	171												
Ki-67 (%), *n* = 6	<14	248	0.23 (−0.17, 0.62)	0.27	79%	0.26 (0.10, 0.43)	**0.002**	62%	−0.07 (−0.44, 0.29)	0.69	78%	−0.06 (−0.22, 0.10)	0.47	0
(10, 17, 26, 28–30)	≥ 14	512												

IDC, invasive ductal carcinoma; DCIS, ductal carcinoma in situ; SMD, standardized mean difference; ADC, apparent diffusion coefficient; D, tissue diffusivity, D^*^, pseudo-diffusivity; f, perfusion fraction; I^2^, inconsistency index; ER, estrogen receptor; PR, progesterone receptor; HER2, human epidermal growth factor receptor; n, number of included studies. The values in bold represent statistical significance.

### Diagnostic Performance

**Figure 7:**
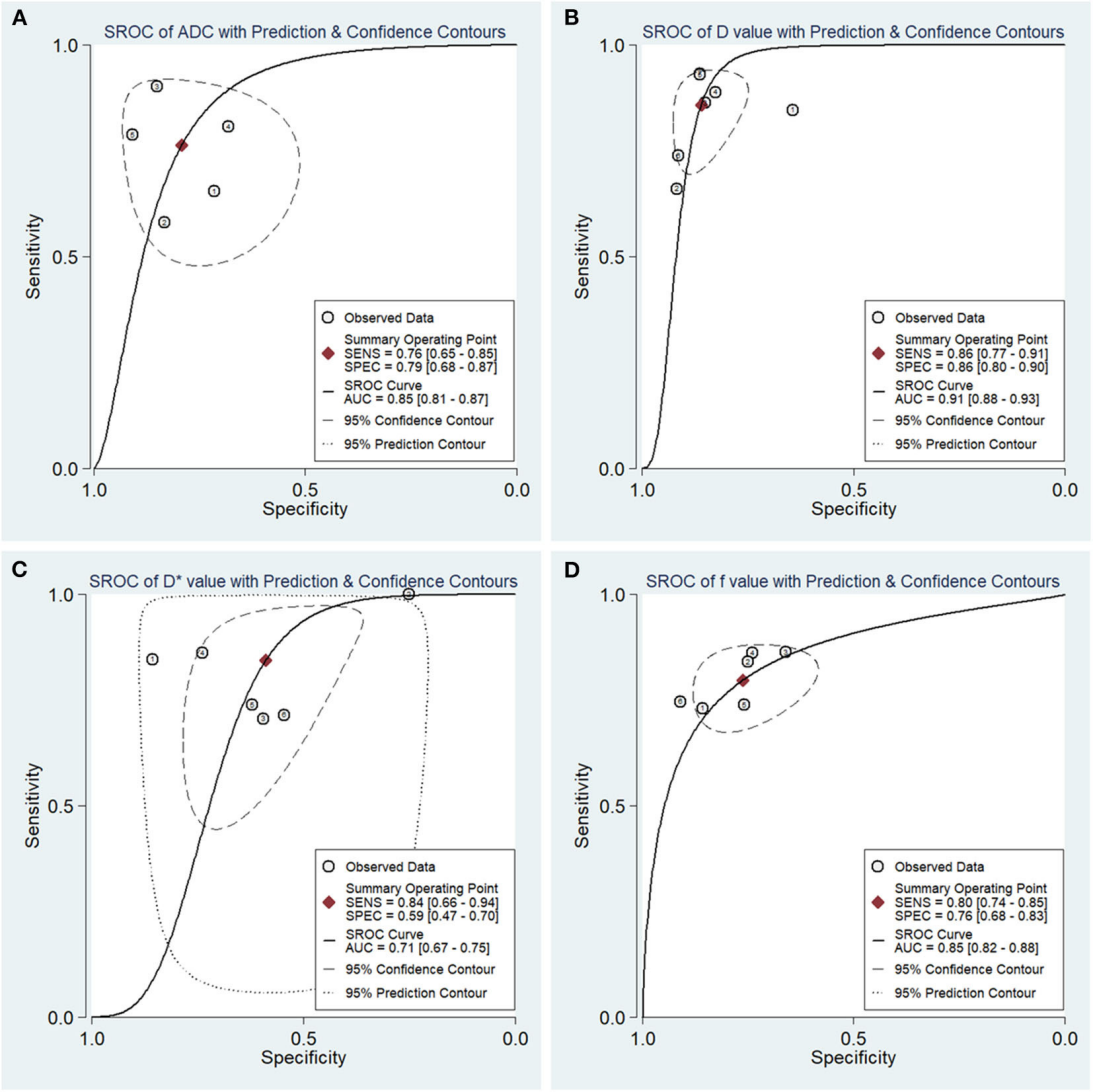
Summary receiver operating characteristic (SROC) curves of **(A)** the apparent diffusion coefficient (ADC), **(B)** tissue diffusivity (D), **(C)** pseudodiffusivity (D*), and **(D)** the perfusion fraction (f) in the diagnosis of breast lesions. D had the largest area under the curve (AUC) among the four parameters, followed by the ADC, f and D* values.

The diagnostic performance as assessed by pooling sensitivity, specificity, PLR, NLR, DOR and the AUCs of the ADC, D, D^*^, and f values are listed in Table 4. Deek's funnel plots and asymmetry tests indicated no obvious publication bias for the *ADC, D*, and f values (*P* = 0.34, 0.28, and 0.21) but potential publication bias for the D^*^ value (*P* = 0.03). Figure 7 plots the SROC curves of the ADC, D, D^*^ and f values. Because not all the studies reported the diagnostic performance of IVIM-DWI in the detection of breast tumors, there were a small number of studies included for analysis in Table 2 and Figure 7. The D value demonstrated good diagnostic performance (sensitivity = 86%, specificity = 86%, AUC = 0.91) in the differential diagnosis of breast tumors, which was comparable to that of the ADC (sensitivity = 76%, specificity = 79%, AUC = 0.85), followed by the f (sensitivity = 80%, specificity = 76%, AUC = 0.85) and D^*^ values (sensitivity = 84%, specificity = 59%, AUC = 0.71).

**Table 4:** Pooled estimates and heterogeneity measures for the ADC, D, D^*^ and f values.

**Indicators**	**Sensitivity**	**Specificity**	**PLR**	**NLR**	**DOR**	**AUC**	**I**^****2****^	
							**Sensitivity %**	**Specificity %**
ADC	0.76 (0.65, 0.85)	0.79 (0.68, 0.87)	3.7 (2.2, 6.0)	0.30 (0.19, 0.48)	12 (5, 30)	0.85 (0.81, 0.87)	76.66	38.87
D	0.86 (0.77, 0.91)	0.86 (0.80, 0.90)	6.1 (4.4, 8.6)	0.17 (0.10, 0.26)	37 (21, 67)	0.91 (0.88, 0.93)	79.59	19.14
D*	0.84 (0.66, 0.94)	0.59 (0.47, 0.70)	2.1 (1.6, 2.6)	0.26 (0.12, 0.56)	8 (3, 18)	0.71 (0.67, 0.75)	79.84	61.72
f	0.80 (0.74, 0.85)	0.76 (0.68, 0.83)	3.4 (2.4, 4.6)	0.27 (0.21, 0.35)	13 (8, 20)	0.85 (0.82, 0.88)	15.09	16.32

ADC, apparent diffusion coefficient; D, tissue diffusivity, D^*^, pseudo-diffusivity; f, perfusion fraction; PLR, positive likelihood ratio; NLR, negative likelihood ratio; DOR, diagnostic odds ratio; AUC, area under the curve; I^2^, inconsistency index.

### Posttest Probabilities

**Figure 8:**
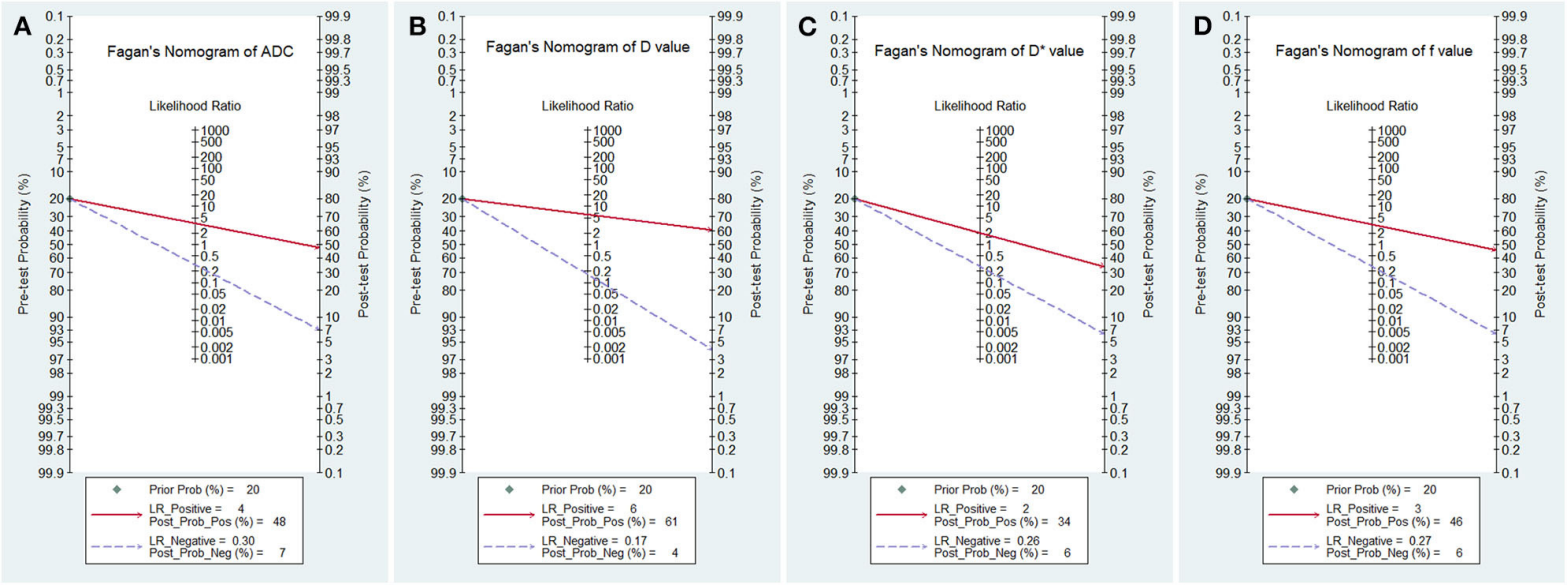
Fagan's nomogram of **(A)** the apparent diffusion coefficient (ADC), **(B)** tissue diffusivity (D), **(C)** pseudodiffusivity (D*), and **(D)** the perfusion fraction (f).

The likelihood ratio and post-test probability are also important for diagnosing a disease (31), which estimated whether a patient was diagnosed with a certain disease using the MRI parameters. Figure 8 plotted the Fagan's nomograms of the ADC, D, D^*^, and f values for predicting post-test probabilities. All the pre-test probabilities were set at 20% by default. We regarded the diagnosis of breast cancer as a positive event, corresponding to a higher f value and lower *ADC* and D values. Similarly, diagnosing benign lesions with a lower f value and higher *ADC* and D values represented a negative event. From a pre-test probability of 20%, the post-test probability increased to 48% with a PLR of 3.7 and decreased to 7% with an NLR of 0.30 based on the ADC. This indicated that the diagnostic probability for breast cancer will be obviously enhanced in cases with a lower ADC than in cases without an ADC measurement. By contrast, the probability of a breast cancer diagnosis will significantly drop from 20 to 7% when a negative event occurs (e.g., a higher ADC). Similarly, when using D for predicting a diagnosis, the post-test probability of diagnosing breast cancer will reach 61% with a PLR of 6.1 and drop to 4% with an NLR of 0.17. The inclusion of f increases the post-test probability of diagnosing breast cancer to 46% with a PLR of 3.4 and decreases it to 6% with an NLR of 0.27. These data indicated that IVIM parameters, especially the D value, increased the accuracy for diagnosing breast cancer.

## Discussion

IVIM-DWI is a non-invasive technique that shows superiority in reflecting tumor cellularity and perfusion without the need for contrast agent. It has already been applied in the differentiation of lung nodules (32), thyroid nodules (33), prostate (34) and brain tumors (35) with good diagnostic performance. Although IVIM has become a research hotspot in whole-body tumors, especially in breast tumors, to the best of our knowledge, there is still no study on breast tissues with a sufficient sample size to establish the value of IVIM for quantitatively distinguishing breast cancer from benign lesions and their molecular subtypes. Our study provides a timely summary of this issue by pooling all published evidence with strict inclusion criteria and quality assessments. The results showed a promising prospect for incorporating IVIM-DWI into MRI protocols for the breast.

In our study, the SMDs suggested that malignant breast tumors demonstrated lower *ADC* and D values and higher f values than did benign lesions. Breast cancer usually has dense cellularity with a high capacity for proliferation, which may reduce the extracellular space and limit the diffusion of water molecules thus causing a reduction in the diffusion coefficient. The pooled results also suggested that the D value improved the diagnostic performance with a slightly higher sensitivity, specificity, AUC, DOR and post-test probability than conventional ADC. Theoretically, the monoexponential model may miscalculate the water molecule movement in conjunction with microcirculation perfusion and therefore overestimate the ADC value (14). The D value can precisely calculate the true diffusion without the influence of perfusion-related diffusion (15), but a larger number and higher *b*-value applied in the IVIM model will significantly prolong the scanning times and introduce motion and susceptibility artifacts.

Interestingly, malignant breast tumors demonstrated a significantly higher f value but a non-significantly higher D^*^ value than did the benign lesions. This mainly arose from increased angiogenesis in breast cancer (14). The f value also demonstrated a higher specificity of 0.76 and an AUC of 0.85 compared with the specificity of 0.59 and AUC of 0.71 for the D^*^ value. In addition, the mean D^*^ values of breast cancer ranged from 3.85 to 109.78 × 10^−3^ mm^2^/s with a huge SD among the included studies, which indicated that the D^*^ value was not robust and could not further increase the diagnostic sensitivity and specificity; however, the f value was able to more accurately reflect tissue perfusion. Liu et al. (14) also stated that the D^*^ value may be unreliable in the IVIM model due to the low signal-to-noise ratio and the poor measurement reproducibility.

The pooled results indicated lower ADC and D values in IDC than in DCIS, suggesting denser cellularity and a more limited extracellular volume fraction in IDC with more aggressive features (7). Therapeutic strategies and treatment efficacy are closely related to intrinsic biological subtypes of breast cancer (17). Our pooled results suggested that the lesions with metastatic lymph nodes had higher D^*^ and f values than did lesions without lymph node metastasis. The IVIM model provides a surrogate marker for predicting lymph node status, and rich tumor perfusion owing to neovascularization may facilitate lymphatic metastasis (36). The results also suggested greater tumor perfusion (D^*^ and f value) in HER2-positive cancer. HER2 is an important prognostic factor of breast cancer and is closely correlated with tumor proliferation, invasion and metastasis. It can promote tumor angiogenesis and lymphangiogenesis via regulation of vascular endothelial growth factor (VEGF) in breast cancer and therefore improve tumor perfusion (10). Our study also suggested that breast cancer with high Ki-67 expression has a significantly lower D value (*P* = 0.002) instead of ADC value (*P* = 0.27), which was mainly due to active proliferation and a higher cell density. The results suggested that the D value better reflected Ki-67 status than did ADC values when assessing the cell density and proliferation status. Our results also suggested a significant difference in the perfusion-related parameters (D^*^) between ER or PR statuses. The detection of ER and PR is of great significance for estimating the prognosis of breast cancer and guiding endocrine therapy, as patients with positive ER and PR expression showed high responsiveness to hormone therapies. Previous studies have reported that positive ER and PR expression inhibited tumor angiogenesis by decreasing the level of VEGF (7, 10, 37, 38), which leads to lower D^*^ value in ER- and PR-positive tumors.

The correlation results suggested no significant threshold effects in the ADC (*r* = −0.100, *P* = 0.873), D (*r* = 0.342, *P* = 0.452), D^*^ (*r* = −0.029, *P* = 0.957) and f values (*r* = 0.829, *P* = 0.524); thus, they are not the main contributors to the heterogeneity. The ADC, D, D^*^, and f values all demonstrated obvious heterogeneity, which should be further explored. First, most of the included studies did not control for age or menstrual cycle for analysis, which may have introduced heterogeneity. Second, 1.5T and 3.0T MR scanners with various combinations of *b*-values were used to perform IVIM-DWI in these studies, which may influence the accuracy of the calculations of diffusion and perfusion coefficients. Third, the post-processing methods were different, as some studies (9, 26) performed histogram analyses for the whole lesions, while the others assessed the lesions at the largest section as the region of interest. Last, the tumor subtypes were inconsistent in the malignant and benign groups; this may result in different biological characteristics and consequent variations in the IVIM values.

There were several limitations to this meta-analysis. First, the small number of studies regarding the histological/molecular subtypes of breast cancer was still insufficient to draw a robust conclusion. Second, we did not perform a horizontal comparison with other diffusion imaging techniques, such as diffusion tensor imaging and diffusion kurtosis imaging, both of which provide information that reflects directional characteristics and tissue complexity. A combination of these sequences may further improve the specificity in characterizing breast lesions.

## Conclusions

IVIM-DWI parameters were adequate and superior to the ADC in differentiating breast tumors. They can further differentiate IDC from DCIS. Besides, IVIM-derived parameters also showed unique superiority in identifying lymph node metastasis, histologic grade, and hormone receptor, and HER2 and Ki-67 status. It is quite suitable when making treatment plans and prognosis assessments.

## Data Availability Statement

All datasets generated for this study are included in the article/supplementary material.

## Author Contributions

NH and YW conceived the study and revised the manuscript. JL and SZ drafted the manuscript. ZL and YK searched the databases and acquired the data. TM, JC, and CZ performed data analysis and interpretation. All authors contributed substantially to the preparation of the manuscript.

## Conflict of Interest

The authors declare that the research was conducted in the absence of any commercial or financial relationships that could be construed as a potential conflict of interest.

## Abbreviations

AUC: area under the curve
ADC: apparent diffusion coefficient
D: tissue diffusivity
D^*^: pseudodiffusivity
ER: estrogen receptor
f: perfusion fraction
HER2: human epidermal growth factor receptor 2
IVIM-DWI: intravoxel incoherent motion–diffusion–weighted imaging
SMD: standardized mean difference
I^2^: inconsistency index
PR: progesterone receptor.
